# Biomarkers of oxidative stress and inflammation in urine samples of extremely preterm newborns and risk for autism at corrected-age 24 months

**DOI:** 10.1192/j.eurpsy.2025.652

**Published:** 2025-08-26

**Authors:** T. Bollain Muñoz, J. Merchán-Naranjo, B. Almansa, C. Chafer, M. Vento, D. Blanco-Bravo, A. García-Blanco, L. Pina-Camacho

**Affiliations:** 1 Psychiatry, HGUGM; 2 IISGM; 3 Universidad Complutense; 4 CIBERSAM, Madrid; 5 IISLAFE, Valencia; 6 Neonatology, HGUGM, Madrid, Spain

## Abstract

**Introduction:**

Extremely preterm birth (defined as birth before 28 weeks’ gestational age) has been associated with an increased risk of developing autism spectrum disorder (ASD) in infancy. The underlying pathophysiological mechanisms driving the emergence of ASD in these children remain unknown, although growing evidence suggests that oxidative stress and inflammation play important roles.

**Objectives:**

To detect if urinary protein oxidation could serve as a non-invasive early predictor of ASD risk in extremely preterm newborns.

**Methods:**

In two Spanish cohorts recruited between 2020 and 2022, consisting of 76 extremely preterm newborns, we collected urine samples from birth up to the first week of life (T1 = birth, T2 = 24–72 hours, T3 = day 7) and analyzed biomarkers of oxidative stress and inflammation. We assessed the risk for ASD at a corrected age of 24 months. Using liquid chromatography-mass spectrometry, we measured levels of lipid peroxidation, DNA and protein oxidation metabolites, along with markers of inflammation. We investigated the association between longitudinal urine marker levels and the primary outcome (risk for ASD, defined as an MCHAT-R/F score ≥ 2 at 24 months).

**Funding:**

The study was supported by the Spanish Ministry of Science & Innovation, Instituto de Salud Carlos III (grant numbers PI17/01997, PI18/01352, PI20/01342, PI23/00368); co-financed by ERDF Funds from the European Commission, “A way of making Europe”, Fundación Familia Alonso & Fundación Alicia Koplowitz.

**Results:**

Thirty-two of the 76 patients completed the 24-month follow-up period and had samples available from at least two of the three time points. Compared to those with no risk for ASD (n = 21), patients at risk for ASD (n = 11) exhibited significantly lower O-tyrosine levels at birth (d = 1.296, p = .048). Moreover, O-tyrosine levels increased significantly over time in at-risk patients relative to non-ASD risk patients from birth to day 7 (p = .032), suggesting protein damage due to significant oxidative stress.

**Conclusions:**

These findings indicate that urinary protein oxidation markers could serve as promising, non-invasive early predictors of ASD risk in extremely preterm newborns.

**Disclosure of Interest:**

None Declared

